# Regulation of prophage induction and lysogenization by phage communication systems

**DOI:** 10.1016/j.cub.2021.08.073

**Published:** 2021-11-22

**Authors:** John B. Bruce, Sébastien Lion, Angus Buckling, Edze R. Westra, Sylvain Gandon

**Affiliations:** 1 Environment and Sustainability Institute, University of Exeter, Penryn Campus, Exeter, UK; 2 CEFE, CNRS, Univ Montpellier, EPHE, IRD, Univ Paul Valéry Montpellier 3, Montpellier, France

**Keywords:** arbitrium, lysis-lysogeny, microbiology, phage, prophage, induction

## Abstract

Many viruses cause both lytic infections, where they release viral particles, and dormant infections, where they await future opportunities to reactivate.^1^ The benefits of each transmission mode depend on the density of susceptible hosts in the environment.^2^, ^3^, ^4^ Some viruses infecting bacteria use molecular signaling to respond plastically to changes in host availability.^5^ These viruses produce a signal during lytic infection and regulate, based on the signal concentration in the environment, the probability with which they switch to causing dormant infections.^5^^,^^6^ We present an analytical framework to examine the adaptive significance of plasticity in viral life-history traits in fluctuating environments. Our model generalizes and extends previous theory^7^ and predicts that host density fluctuations should select for plasticity in entering lysogeny as well as virus reactivation once signal concentrations decline. Using *Bacillus subtilis* and its phage phi3T, we experimentally confirm the prediction that phages use signal to make informed decisions over prophage induction. We also demonstrate that lysogens produce signaling molecules and that signal is degraded by hosts in a density-dependent manner. Declining signal concentrations therefore potentially indicate the presence of uninfected hosts and trigger prophage induction. Finally, we find that conflict over the responses of lysogenization and reactivation to signal is resolved through the evolution of different response thresholds for each trait. Collectively, these findings deepen our understanding of the ways viruses use molecular communication to regulate their infection strategies, which can be leveraged to manipulate host and phage population dynamics in natural environments.

## Highlights

• Phages produce and respond to arbitrium signal during the lytic and lysogenic cycles
• Signaling provides indirect information on the availability of susceptible hosts
• Phages adjust lysogenization and reactivation based on this indirect information
• Phages use distinct signal response thresholds for lysogenization and reactivation

## Graphical abstract

**Figure:**
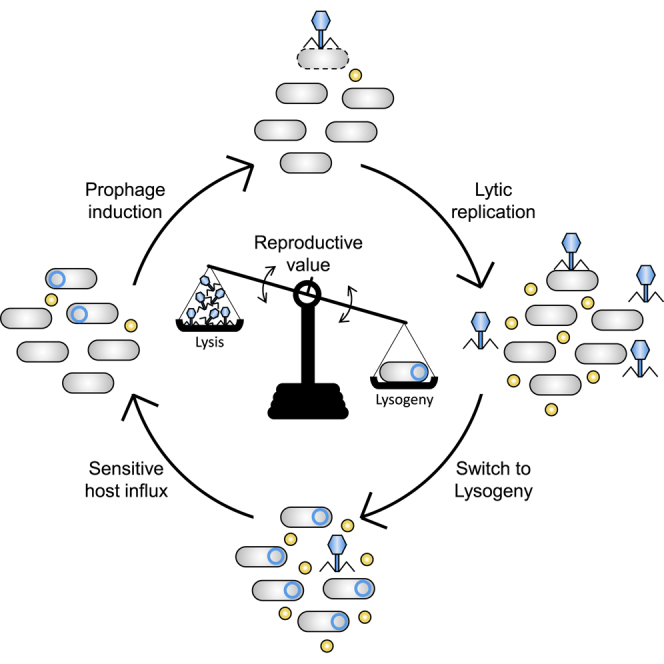

## Teaser

Bruce et al. show that phages use molecular signaling to respond to changing host availability during lytic growth and as prophage. The virus uses different signal response thresholds for the transition to lysogeny versus for reactivation, allowing phages to avoid lysogeny when susceptible hosts are many and induction when they are absent.

## Results and discussion

When susceptible hosts are plentiful, lytic phage replication maximizes the spread of the virus through the host population, whereas lysogeny allows continued replication at each cell division, even in the complete absence of available hosts.^8^, ^9^, ^10^ The ability to switch between these different infection strategies should therefore be favored when host availability fluctuates, as is, for example, the case over the course of a phage epidemic due to host lysis.^1^, ^2^, ^3^ *Bacillus* phages encoding the arbitrium system respond to molecular signals produced during recent infections, switching from lytic to lysogenic replication when the likelihood of finding and successfully infecting another susceptible host is diminished. To examine analytically when this signaling-dependent plasticity in the lysis-lysogeny decision is adaptive, we constructed a mathematical model for the dynamics of a temperate phage that produces and responds to signaling peptides. We use this model to establish when temperate phages should evolve to respond to changes in signal concentration, and whether they should not only regulate the transition from lysis to lysogeny but also the transition from lysogeny to lysis (prophage induction).^11^

First, we generated an epidemiological model (see STAR Methods for details) of a well-mixed bacterial population made up of susceptible cells, lysogenic cells, and free virus particles. Our model assumes that the influx of susceptible cells may vary with time, and we allow lysogenization and reactivation rates to be functions of the concentration of arbitrium in the environment. This model thus tracks the densities of bacteria (uninfected and lysogens), phages (free phage and lysogens), and signal concentrations. We then use this framework to understand and predict how the response of phages to arbitrium signals will evolve. Specifically, we determine the fate of viral mutants with altered lysogeny or prophage induction in response to changes in signal concentration in a fluctuating environment. This evolutionary analysis shows that the selection for the mutant varies with the availability of susceptible cells in the environment (see STAR Methods for details). Crucially, we show that the direction of selection for lysogeny and for reactivation is governed by the difference between the time-varying reproductive values of the virus in the different states of its life cycle (as a prophage in a lysogen or as a virus particle): when the reproductive value of a prophage is higher than the reproductive value of B virus particles (where B is the burst size of the virus), lysogeny is favored and reactivation is disfavored. This simple and intuitive condition can be used to recover the results of earlier theoretical analysis in constant environments.^10^ But this condition is particularly useful to examine the evolution of the virus in fluctuating environments.^12^ In agreement with a previous model,^7^ this analysis confirms that plasticity in lysogenization induction in response to increases in arbitrium signal concentrations can be adaptive but only when there is fluctuation in host availability in the environment. Crucially, our theory also predicts that it can be adaptive for phage to evolve plasticity in prophage induction in response to decreases in arbitrium signal concentrations but again only when host availability in the environment fluctuates (see STAR Methods for details).

**Figure 1:**
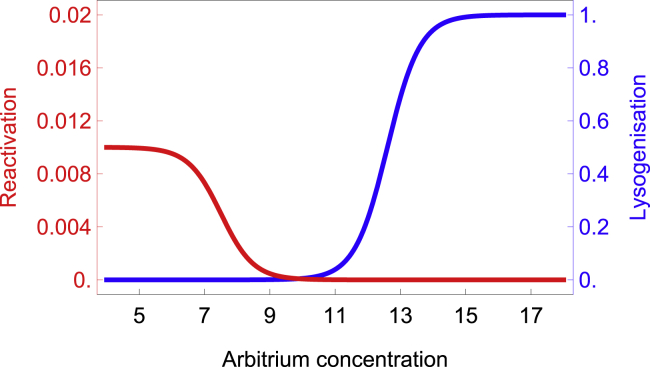
Coevolutionary stable strategies for reactivation and lysogenization In a theoretical evolutionary model (see STAR Methods for details), fluctuating concentrations of signaling peptide select for plasticity in both lysogeny and reactivation. The joint evolution of these two traits is expected to yield very different reaction norms with arbitrium concentration. See also Figures S1–S3.

Given that the model predicts that two traits of the phage, lysogenization and prophage induction, would evolve jointly in response to arbitrium signaling, we analyzed the ultimate coevolutionary outcomes between these traits (see STAR Methods for details). This analysis shows that, in a stable coevolutionary outcome, prophage induction and lysogenization respond in opposite ways to arbitrium, and, crucially, the threshold concentration of arbitrium that triggers induction is much lower than the one that triggers lysogenization (Figure 1). The evolution of diametrically different response thresholds allows phages in either state to switch modes of transmission only when it is most advantageous: infectious phage particles avoid lysogenization at the low signal concentrations experienced when susceptible hosts are likely still available, while prophages avoid induction when signal indicates a high risk of not finding a new host. In addition, the evolution of distinct arbitrium concentration thresholds at which these transitions occur ensures that lysogens are stably maintained across a broader range of arbitrium concentrations.

**Figure 2:**
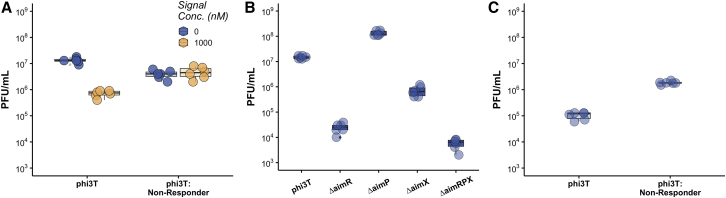
The arbitrium system modulates prophage reactivation (A) Plaque-forming units (PFUs) produced by prophage reactivation from phi3T (wild-type) and phi3T^*aimR*-N202A^ (signal non-responder) lysogens in the presence (1,000 nM) or absence of signaling molecules (12 h growth in LB). (B) phi3T receptor (ΔaimR), signal production (aimR), lysogeny regulator (ΔaimX), and arbitrium system (ΔaimRPX) deletion mutants (18 h growth in LB in the absence of synthetic signal). (C) phi3TΔ*aimP* (signal non-producer) lysogens of hosts carrying the *aimX* gene under the control of a xylose promoter in LB containing 0% xylose (uninduced) or 0.2% xylose (induced). n = 6 in all treatments. Error bars represent standard error.

To experimentally test the model prediction that prophage reactivation is regulated by arbitrium signaling, we first measured prophage induction in the presence or absence of synthetic signal from *Bacillus subtilis* strain 168 lysogenized with phage phi3T, which is one of the best studied models of the arbitrium signaling system.^5^^,^^6^^,^^13^^,^^14^ We cultured phi3T lysogens in LB or LB supplemented with synthetic signaling peptide and quantified prophage reactivation. We found that prophage reactivation was significantly reduced in lysogens exposed to synthetic signal relative to those that were not (F_1,10_ = 104.9; p ≤ 0.0001; Figure 2A). We hypothesized that the genes responsible for regulating prophage induction in response to arbitrium signaling would be the same as those responsible for the regulation of lysogenization in response to arbitrium. The phi3T arbitrium system is composed of 3 genes: *aimR* encodes the signal receptor that activates *aimX* expression in its signal-free form; *aimP* encodes the signal; and *aimX* encodes a non-coding RNA that suppresses lysogeny.^5^^,^^6^^,^^13^ To test our hypothesis, we first repeated the same experiment using phi3T^AimR-N202A^ lysogens, which carry a single amino acid substitution in the signal receptor (*aimR*) that makes it unable to respond to signal.^14^ We found that addition of signaling peptide caused no significant reduction in phi3T^*aimR*-N202A^ lysogens (F_1,10_ = 0.5656; p = 0.469; Figure 2A). These results demonstrate that prophage induction is responsive to the presence of signaling peptides as predicted by our model and that the *aimR* protein is responsible for signal detection in the prophage state.

To test for the involvement of the other arbitrium genes, we generated all possible single-deletion mutants and a triple-deletion mutant and compared reactivation of mutant prophages to wild-type (WT) phi3T lysogens. We found deletion of the signaling system or its constituent parts had a significant impact on prophage reactivation relative to WT phi3T (F_4,25_ = 116.9; p ≤ 0.0001; Figure 2B). Deletion of *aimR*, the peptide receptor, and *aimX*, the negative regulator of lysogeny, reduced reactivation from the prophage state relative to phi3T, as did deleting the entire signaling system. Interestingly, deleting *aimP*, the signal peptide producer, increased prophage reactivation relative to phi3T, suggesting that signaling peptide may be produced by lysogens and this may influence prophage reactivation. These observations are consistent with the previously described roles of *aimR*, *aimP*, and *aimX* in regulating the transition from lytic to lysogenic replication,^5^^,^^6^^,^^13^ with *aimX* acting as the negative regulator of lysogeny. To further corroborate this result, we tested whether *aimX* expression drives reactivation and lysis in the prophage state using ectopic expression of *aimX* from the bacterial host. We found that inducing *aimX* expression from the host significantly increased prophage reactivation relative to uninduced hosts (F_1,10_ = 268.6; p ≤ 0.0001; Figure 2C). Collectively, these results demonstrate that the arbitrium signaling genes, *aimPRX*, not only play a role in lysogenization but also in the prophage reactivation process.

**Figure 3:**
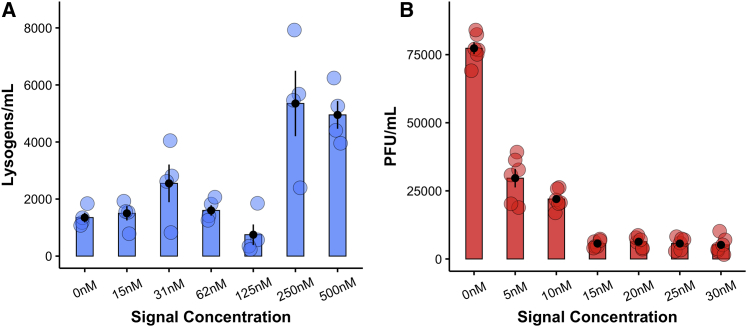
Infection and prophage signal responses (A) Lysogen formation from BEST7003 cultured in LB with increasing concentrations of signaling peptide and infected with phi3TΔ*aimP*(*spc*) at MOI = 0.1 (40-min infection, n = 4). (B) PFUs produced by prophage reactivation from phi3TΔ*aimP*(*spc*) (signal negative) lysogens cultured in LB with increasing concentrations of signaling peptide (8 h growth, n = 6). Error bars represent standard error.

Our model also predicts that phage should evolve different response thresholds for lysogenization and prophage induction (Figure 1). To experimentally test how signal concentrations shape the lysis-lysogeny decision during infection, we resuspended log-phase *Bacillus subtilis* cells in LB media supplemented with synthetic signaling peptide ranging from 0 nM to 500 nM and quantified lysogen formation following infection with a non-signal-producing phage mutant to exclude signal production by the phage as a confounding factor. Consistent with earlier work,^5^ we found that signal concentration significantly impacted lysogen formation (F_6,21_ = 10.55; p = 1.57 × 10^−5^) and that this was driven by significant increases in lysogen formation at 250 nM (t = 4.997; p = 6.03 × 10^−5^) and 500 nM (t = 4.497; p = 0.0002) concentrations of signaling peptide (Figure 3A). Next, we quantified prophage reactivation from non-signal-producing lysogens in LB media supplemented with between 0 nM and 500 nM of signaling peptide. We found that increasing signal concentration decreases prophage reactivation (F_6,35_ = 226.8; p ≤ 2.2 × 10^−16^) and that even 5 nM of signal peptide was enough to significantly decrease prophage reactivation (t = −19.23; p ≤ 2 × 10^−16^; Figure 3B). Together, these results support the predictions of our model, demonstrating that high concentrations of signaling peptide promote the switch to lysogeny during lytic infection and that prophages only revert back to lytic replication at very low concentrations of signal.

**Figure 4:**
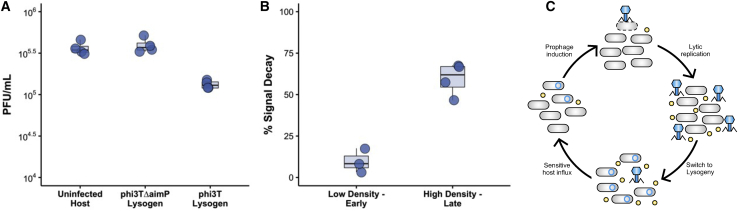
Signal production, durability, and decay (A) Prophage induction in spent media of uninfected BEST7003, phi3TΔ*aimP* lysogens, and phi3T lysogens (n = 4). (B) The durability of signaling peptides was quantified under different conditions: spent media of *Bacillus subtilis* BEST7003 extracted from early (3 h) low-density (∼0.3) cultures and late (8 h) high-density (∼1.8) cultures, supplemented with signaling peptide to 1,000 nM and incubated for 12 h. Signal decay was calculated by comparing initial and final signal concentrations. Signal concentrations were calculated using BEST7003:RPXgfp (a signal reporter containing the phi3T *AimR-AimP-AimX* locus genetically fused to a fluorescent reporter gene) and a calibration curve constructed using spent media supplemented with known concentrations of signaling peptide (n = 4; see STAR Methods for details). Error bars represent standard error. (C) Conceptual model of lysis and lysogeny and prophage induction as a function of signal production and decay. Lysogenic and lytic infections^5^ produce signal that is decayed at high cell densities. At high lysogen densities, constitutive signal production maintains the prophage state. An influx of susceptible cells, or invasion of a susceptible population, rapidly decays signal, triggering prophage induction. Subsequent lytic infections remove susceptible hosts from the population, increasing signal concentrations and triggering the switch to lysogeny. See also Figures S4A and S4B.

Our model predicts, and our experimental work confirms, that prophage reactivation is responsive to the presence of signaling peptides. Yet what information is provided by the signal is unclear and will depend on the dynamics of signal production and decay.^15^ In the model, we assumed that infected cells produce signal during both lytic^5^ and lysogenic replication and that signal decay is dependent on the total host density. Under those assumptions, the net signal concentration provides a measure of the epidemiological status of the population. Consistent with those ideas, we observed that deletion of *aimP*, the signal peptide producer, increased prophage reactivation relative to phi3T, suggesting that signaling peptide may be produced by lysogens and this may influence prophage reactivation (Figure 2B). To test this hypothesis, we quantified prophage induction in lysogens grown in the spent media from unlysogenized BEST7003 cultures and phi3T and phi3TΔ*aimP* lysogens (Figure 4A). We found that spent media from phi3T lysogens significantly reduced prophage reactivation relative to spent media from uninfected BEST7003 (t = −5.21; p = 0.0006), whereas prophage induction in spent media from signal non-producer phi3TΔ*aimP* was not significantly different (t = 0.728; p = 0.484). As these lysogens differed only in the presence or absence of the signal peptide producer gene *aimP*, these results suggest that lysogenic as well as lytic^5^ infections contribute to signal production.

To explore the durability of the signaling peptide, we quantified signal decay across different environmental conditions. We supplemented LB media with 1,000 nM signaling peptide and quantified signal concentration over time in the absence of bacteria. We found no significant decrease in signal concentration over 72 h (F_1,7_ = 5.483 × 10^−28^; p = 1; Figure S4A). These data corroborate previous anecdotal evidence that signal is durable and suggest that the signaling peptide is unlikely to act solely as an indicator of recent infections.^5^ To investigate whether signal can be decayed by the activity of hosts, we grew *Bacillus subtilis* in LB media supplemented with signaling peptide to 1,000 nM and quantified the signal concentration of spent media extracted after 18 h of growth. To explore potential mechanisms of signal degradation, we used both the *Bacillus subtilis* strain 3610 and the isogenic 3610Δ*oppD* mutant, which carries a loss-of-function mutation in the oligopeptide permease ABC transporter that is responsible for internalizing the signaling peptide.^16^ We found that both strains decayed the signal and that there was no significant difference in the decay caused by either strain (F_1,4_ = 0.03552; p = 0.8597; Figure S4B). These results do not rule out the possibility that signal peptides are decayed intracellularly but suggest that extracellular decay does occur.

To determine whether signal can be decayed by secreted extracellular products, we extracted spent media from *Bacillus subtilis* after 3 h (relatively low density) and after 8 h growth (relatively high density) in LB media and quantified signal degradation caused by the spent media. We found that high-density spent media caused significant signal decay (t = −12.111; degree of freedom [df] = 3; p = 0.0012), whereas spent media from low-density cultures did not (t = −1.2244; df = 3; p = 0.3082; Figure 4B). These results confirm that the signaling peptide produced by phi3T is decayed by extracellular products produced by its *Bacillus subtilis* host and that decay is host density dependent.

Our model predicts that prophages should avoid induction when signal is present, as lysis is only advantageous when susceptible hosts are available. The production of signaling peptide by lysogens and the decay of signal at high host densities provides a potential mechanism by which prophages can gauge whether they are surrounded by susceptible or lysogenized hosts and adjust their transmission strategy accordingly. Although the mechanism of signal decay remains to be determined, it is possible that this is caused by secreted proteases that are produced by *Bacillus subtilis*. A major serine protease, and a metalloprotease, encoded by the *apr* and *npr* genes, respectively, have been reported to account for ∼95% of its extracellular protease activity.^17^ These secreted products are both indirectly regulated by the comX quorum-sensing system and are capable of degrading signaling peptides.^18^^,^^19^ Due to their indirect regulation by the comX quorum-sensing system, extracellular proteases are produced and secreted mostly at high cell densities.^18^^,^^19^ Our data suggest that, if lysogenized cells make up a minority of a dense host population, signal concentrations will rapidly decline, triggering prophage reactivation. Conversely, constitutive signal production by lysogens may prevent reactivation when prophages are surrounded by already lysogenized hosts. In this way, prophage induction would be limited to conditions that correlate with the availability of high densities of susceptible hosts (Figure 4C). Evidence that dormant viruses and other mobile genetic elements infer information on host availability from their environment is becoming increasingly common: multiple temperate phages are known to monitor their hosts’ quorum-sensing systems in order to optimize their lysis-lysogeny decisions,^20^, ^21^, ^22^, ^23^, ^24^ while the integrative and conjugative element ICEBs1 of *Bacillus* also uses its own Rap/Phr signaling system to limit excision and transfer until the host is surrounded by a high density of cells lacking ICEBs1.^25^

We have shown, theoretically and experimentally, that temperate phages use the arbitrium molecular signaling system to optimize both lysis-lysogeny and prophage induction decisions. By responding to the concentration of signal in the environment, and using different response thresholds for lysogeny and reactivation, they are able to choose the transmission strategy that maximizes the number of new infections in a given environment. Our results add to growing evidence that temperate viruses use biotic and abiotic cues to modulate their infection strategies and that this plasticity is evolutionarily beneficial in uncertain environments.

## STAR★Methods

### Key resources table

**Table:** 

REAGENT or RESOURCE	SOURCE	IDENTIFIER
**Bacterial and virus strains**		

Bacillus subtilis BEST7003	Bacillus Genetic Stock Centre (BGSC)	N/A
Bacillus subtilis 3610	Bacillus Genetic Stock Centre (BGSC)	N/A
Bacillus subtilis 3610ΔoppD	Bacillus Genetic Stock Centre (BGSC)	N/A
Bacillus subtilis BEST7003:RPXgfp	Gift from Rotem Sorek	N/A
Bacillus subtilis BEST7003:aimX	Erez et al., 2017^5^	N/A
Phi3T	Bacillus Genetic Stock Centre (BGSC)	N/A
phi3TΔaimR	This work	N/A
phi3TΔaimP	This work	N/A
phi3tΔaimX	This work	N/A
phi3TΔaimRΔaimPΔaimX	This work	N/A
phi3T-AimR-N202A	This work	N/A
phi3TΔaimP(spc)	Erez et al., 2017^5^	N/A
E.coli DH5a	New England Biolabs	N/A

**Chemicals, peptides, and recombinant proteins**		

SAIRGA peptide (98% purity)	Peptide 2.0	N/A
LB Media	Formedium	cat#LMM1
LB Agar	Formedium	cat#LMM02
MnCl2	Sigma-Aldrich	cat#244589
MgCL2	Sigma-Aldrich	cat#M8266
Spectinomycin	Sigma-Aldrich	cat*#S4014*
Kanamycin	Sigma-Aldrich	cat#B5264
Mannose	Sigma-Aldrich	cat#M6020
Xylose	Sigma-Aldrich	cat#X1500
BsaI_HFv2	New England Biolabs	cat#R3733S
SfiI	New England Biolabs	cat#M8266

**Critical commercial assays**		

NEB HIFI Assembly Kit	New England Biolabs	cat#E2621S

**Deposited data**		

All experimental data	This paper	(https://doi.org/10.5061/dryad.mpg4f4r07).

**Oligonucleotides**		

See Table S2.	This paper	N/A

**Software and algorithms**		

R	R Development Core Team^26^	N/A

### Resource availability

#### Lead contact

Further information and requests for resources and reagents should be directed to and will be fulfilled by the lead contact, Edze Westra (E.R.Westra@exeter.ac.uk).

#### Materials availability

This study did not generate new unique reagents.

### Experimental model and subject details

*Bacillus subtilis* 168, BEST7003, 3610 and 3610ΔoppD were obtained from the *Bacillus* Genetic Stock Centre (BGSC). All phage used in this study are derivatives of the wild-type phi3T, also obtained from the BGSC. phi3TΔ*aimP*(spc) (a mutant replacing the *aimP* gene with a spectinomycin resistance cassette^5^), *Bacillus subtilis* BEST7003:*aimX* (strain expressing *aimX* under the control of a xylose promoter^5^), and *Bacillus subtilis* BEST7003:RPXgfp (a signal reporter containing the phi3T *AimR-AimP-AimX* locus genetically fused to a fluorescent reporter gene (gfp) and inserted into the *amyE* locus) were obtained from Rotem Sorek at the Weizmann Institute of Science. All infections were carried out LB media. Strains were cultured in either 6ml of LB media in a 30ml glass universal vial or 1.5ml of LB media in a 24-well plate at 37°C and shaking at 200rpm. Antibiotics were used as follows unless otherwise stated: kanamycin (5 μg/mL) and spectinomycin (100μg/mL). All bacterial and bacteriophage strains used are listed in the Key resources table.

### Method details

#### Epidemiological Model

We model the epidemiological dynamics of a well-mixed population of bacteria infected by a temperate phage to track the density of susceptible cells,S(t), lysogenic cells, L(t), and free virus particles V(t) (see Table S1 for the list of dynamical variables and parameters of the model). We assume there is a temporally variable influx θ(t) of susceptible cells in the bacterial population. Both susceptible and lysogenic cells have a per-capita birth rate r(1−κN(t)), where N(t)=S(t)+L(t) is the total density of the bacterial population and κ measures intraspecific competition. Susceptible and lysogenic cells have a per-capita death rate d. Free virus particles adsorb to bacterial cells at rate a and they suscessfully infect susceptible cells with probability b. Successful infection may either result in lysogenisation of the cell with probability φ, or, with probability 1−φ, to lysis. Upon lysis, B virus particles are produced and virus particles have a per-capita death rate $d_{V}$. Lysogenic bacteria may also produce virus particles when the prophage reactivates at rate α and induces lysis. This yields the following dynamical equations (we drop dependence to time below for readability):

$$\dot{S}=\theta +rS\left(1-\kappa N\right)-\left(abV+d\right)S$$

$$\dot{L}=rL\left(1-\kappa N\right)+ab\varphi VS-\left(\alpha +d\right)L$$

$$\dot{V}=ab\left(1-\varphi \right)BVS+\alpha BL-\left(aN+d_{V}\right)V$$

Crucially we allow the lysogenisation and reactivation rates to be functions of the concentration A of arbitrium in the environment (φ(A) and α(A), respectively). Arbitrium is produced upon phage lysis at rate $\pi_{V}$ and by lysogens at rate $\pi_{L}$. Arbitrium degrades at a constant rate $d_{A}$ but also because of the uptake by bacterial cells in the environment, which yields:

$$\dot{A}=\pi_{V}abVS+\pi_{L}L-\left(d_{A}+\delta N\right)A$$

Let us assume that the influx of susceptible cells is constant: $\theta \left(t\right)=\theta_{0}$. The condition for a resident virus (with phenotypic traits φ(0) and α(0)) to generate an epidemic can be derived from the calculation of the basic reproductive ratio $R_{0}$ using the next-generation matrix method.^2^^,^^27^ Note that arbitrium is absent at this early stage of the epidemic because the concentration of arbitrium builds up only after the successful emergence of the virus. The parasite life-cycle can be decomposed into the production of new lysogenic bacteria (matrix F) and a matrix that captures all the other terms including mortality and transition to the free virus stage (matrix V):

$$F=\left(\begin{array}{ll} r\left(1-\kappa S_{0}\right) & ab\varphi \left(0\right)S_{0}\\ \alpha \left(0\right)B & ab\left(1-\varphi \left(0\right)\right)BS_{0} \end{array}\right)$$

$$V=\left(\begin{array}{ll} \alpha \left(0\right)+d & 0\\ 0 & aS_{0}+d_{V} \end{array}\right)$$

where $S_{0}=\left(r-d+\sqrt{{\left(r-d\right)}^{2}+4r\kappa \theta_{0}}\right)/2r\kappa$ is the equilibrium density of susceptible bacteria before the introduction of the virus. The matrix F gives the rates at which new individuals appear in the provirus or in the free virus stages. The matrix V gives the rate at which these individuals die. The basic reproduction ratio is the spectral radius of $FV^{-1}$ which yields:

$$FV^{-1}=\left(\begin{array}{ll} X & Z\varphi \left(0\right)\\ YB & Z\left(1-\varphi \left(0\right)\right)B \end{array}\right)$$

with:

$$X=\frac{r\left(1-\kappa S_{0}\right)}{\alpha \left(0\right)+d}$$

$$Y=\frac{\alpha \left(0\right)}{\alpha \left(0\right)+d}$$

$$Z=\frac{abS_{0}}{aS_{0}+d_{V}}$$

The basic reproduction rate of the virus is:

$$R_{0}=\left(T+\sqrt{T^{2}-4D}\right)/2$$

with T and D are the trace and determinant of $FV^{-1}$. There are other ways to partition the transitions between classes and alternative derivation of $R_{0}$ can help disentangle the relative contribution of horizontal and vertical transmission rates of the virus^3^^,^^8^^,^^9^.

The above expression of $R_{0}$ can be readily used to find that $R_{0}>1$ (i.e., virus can generate an epidemic in a fully naive population) when T−D>1 which also yields:

$$\frac{abS_{0}}{aS_{0}+d_{V}}>\frac{1}{\varphi \left(0\right)+\left(1-\varphi \left(0\right)\right)\frac{\alpha \left(0\right)}{\alpha \left(0\right)+d-r\left(1-\kappa S_{0}\right)}}$$

Fluctuations in the environment can affect the ability of pathogens to invade fully naive populations^28^, ^29^, ^30^. In the absence of pathogens, the host populations will settle on a periodic attractor. A full analysis of the stability of this disease-free attractor is beyond the scope of this paper. In the remainder of this analysis, we assume that, after successful invasion of the pathogen, the host-pathogen interaction reaches an endemic attractor characterized by periodic fluctuations, and we ask how these fluctuations may affect the evolution of pathogen traits.

#### Evolutionary Model

To understand and predict life-history evolution we need to determine the fate of viral mutations that affect the shape of the functions (i.e., reaction norms) φ(A) and α(A). We thus have to determine the growth rate of a mutant after its appearance in a viral population dominated by a wild-type genotype. Since the virus may appear in two distinct states, a prophage in lysogenic bacteria (L) or a virion outside the cell (V), we can use the following matrix to describe the dynamics of the mutant^12^:

$$R_{m}=\left(\begin{array}{ll} r\left(1-\kappa N\right)-\left(\alpha_{m}\left(A\right)+d\right) & ab\varphi_{m}\left(A\right)S\\ \alpha_{m}\left(A\right)B & ab\left(1-\varphi_{m}\left(A\right)\right)BS-\left(aN+d_{V}\right) \end{array}\right)$$

where the coefficients $r_{ij}$ of the matrix $R_{m}$ refer to the transition between a mutant virus in state i to a new state j. These transitions depend on the birth and death rates of the bacteria but also on virus life-history strategies (lysogenisation and reactivation).

The selection on the mutant at time t is determined by the instantaneous selection gradient^10^^,^^12^:

$$S\left(t\right)=\underset{i}{\sum }\underset{j}{\sum }v_{i}\left(t\right){\frac{\partial r_{ij}\left(t\right)}{\partial z_{m}}|}_{z_{m}=z}f_{j}\left(t\right)$$

where $v_{i}\left(t\right)$ is the individual reproductive value of a virus in class i and $f_{j}\left(t\right)$ is the frequency of the virus in class j. In other words, this gradient measures the influence of a variation of the life-history trait $z_{m}$ induced by the mutation on one component of fitness ($r_{ij}$) weighted by the “quantity” of the virus in class j and the “quality” of class i. This instantaneous selection gradient can help us to understand the fluctuations of selection in temporally variable environments and thus to study the evolution of viral plasticity.

The dynamics of class frequencies is given by:

$${\dot{f}}_{L}=f_{L}\left(t\right)\left(r\left(1-\kappa N\right)-\left(\alpha \left(A\right)+d\right)\right)+f_{V}\left(t\right)ab\varphi \left(A\right)S-\bar{r}\left(t\right)f_{L}\left(t\right)$$

$${\dot{f}}_{V}=f_{L}\left(t\right)\alpha \left(A\right)B+f_{V}\left(t\right)\left(ab\left(1-\varphi \left(A\right)\right)BS-\left(aN+d_{V}\right)\right)-\bar{r}\left(t\right)f_{V}\left(t\right)$$

where $\bar{r}\left(t\right)=\underset{i}{\sum }\underset{j}{\sum }r_{ij}\left(t\right)f_{j}\left(t\right)$.

Similarly, the dynamics of individual reproductive values is given by:

$${\dot{v}}_{L}=-v_{L}\left(t\right)\left(r\left(1-\kappa N\right)-\left(\alpha \left(A\right)+d\right)\right)-v_{V}\left(t\right)\alpha \left(A\right)B+\bar{r}\left(t\right)v_{L}\left(t\right)$$

$${\dot{v}}_{V}=-v_{L}\left(t\right)ab\varphi \left(A\right)S-v_{V}\left(t\right)\left(ab\left(1-\varphi \left(A\right)\right)BS-\left(aN+d_{V}\right)\right)+\bar{r}\left(t\right)v_{V}\left(t\right)$$

#### Evolution of lysogenisation

Selection on lysogenisation is driven by the following gradient of selection (where z is the evolving trait that can affect the lysogenisation rate φ):

$$S_{\varphi ,z}\left(t\right)=\left(v_{L}\left(t\right)-Bv_{V}\left(t\right)\right){\frac{\partial \varphi_{m}\left(A\right)}{\partial z_{m}}|}_{z_{m}=z}abS\left(t\right)f_{V}\left(t\right)$$

In other words, selection for lysogenisation may vary with time and the direction of selection is governed by the difference between the reproductive value $v_{L}\left(t\right)$ of a prophage and the reproductive value $v_{V}\left(t\right)$ of each of the B virions produced upon lysis. Figure S1 shows the temporal dynamics of the densities of the bacteria, the density of virus particles and the concentration of arbitrium. It is important to note that the concentration of arbitrium increases when the density of susceptible cells start to drop. In other words, in these conditions the concentration of arbitrium is carrying an indirect information regarding the diminution of the availability of susceptible cells. This is important information that can be used by the virus to optimize the timing of the switch driving the lysis-lysogeny decision.

The long-term evolution of the shape of the reaction norm φ(A) is driven by the integral of this instantaneous selection gradient over a period of the fluctuation of the environment:

$$S_{\varphi ,z}=S_{\varphi ,z}\left(t\right)$$

where $X=\frac{1}{T}\overset{\tau +T}{\underset{\tau }{\int }}X\left(t\right)dt$ is the average over one period (T) of the fluctuation of the environment.

Moving forward in the analysis of the evolution of lysogenisation requires an explicit function $\varphi_{m}\left(A\right)$ and we use:

$$\varphi_{m}\left(A\right)=\varphi_{0m}\left(1-p_{m}\right)+p_{m}F_{m}\left(A\right)$$

with

$$F_{m}\left(A\right)=\varphi_{max}/\left(1+{\text{e}}^{-\lambda_{\varphi }\left(A\left(t\right)-A_{\varphi }\right)}\right)$$

This function allows us to consider lysogenisation as a fixed strategy $\varphi_{0}$ when p=0 (no plasticity) or as a conditional function (plastic trait) where $\varphi_{max}$ is the maximal value of $F_{m}\left(A\right)$, $A_{\varphi }$ is the value of A where $F_{m}\left(A\right)=\varphi_{max}/2$ and $\lambda_{\varphi }$ is the slope of the function $F_{m}\left(A\right)$ when $A=A_{\varphi }$.

Let us first consider the evolution of a fixed lysogenisation strategy (when p=0) in a constant environment. When the influx of susceptible bacteria does not vary with time the selection on the trait $\varphi_{0}$ is given by $S_{\varphi ,\varphi_{0}}$:

$$S_{\varphi ,\varphi_{0}}\propto \left({\hat{v}}_{L}-{\hat{v}}_{V}B\right)ab\hat{S}{\hat{f}}_{V}$$

The sign of $S_{\varphi ,\varphi_{0}}$ is governed by the sign of $\left({\hat{v}}_{L}-{\hat{v}}_{V}B\right)$, where the hat symbol refers to the value of the dynamical variables at this endemic equilibrium. We thus need to determine the reproductive values at this endemic equilibrium.

If $R_{0}>1$ the system reaches an endemic equilibrium where $\bar{r}\left(t\right)=0$ and all the dynamical variables are fixed. The frequencies and the reproductive values are also fixed and we can use ${\dot{v}}_{L}=0$ to show:

$$\left({\hat{v}}_{L}-{\hat{v}}_{V}B\right)=\left(r\left(1-\kappa \hat{N}\right)-d\right)\frac{{\hat{v}}_{L}}{\alpha \left(\hat{A}\right)}$$

In other words the sign of ${\hat{v}}_{L}-{\hat{v}}_{V}B$ is given by the sign of $r\left(1-\kappa \hat{N}\right)-d$.

Since $\dot{S}=0$ we know that:

$$r\left(1-\kappa \hat{N}\right)-d=ab\hat{V}-\theta_{0}/\hat{S}$$

The above expression means that if $\theta_{0}=0$ then ${\hat{v}}_{L}-{\hat{v}}_{V}B>0$. In other words, selection favors mutations that increase the rate of lysogenisation toward a maximal value of φ. Hence we recover the result of Wahl et al.^10^ who showed that evolution toward an intermediate level of lysogeny (i.e., evolutionary stable φ<1) requires an influx of susceptible hosts. In our model we find that this influx of susceptible cells has to be higher than a threshold: $\theta_{0}>\;ab\hat{V}\hat{S}$.

Could an evolutionary stable fixed strategy be invaded by a plastic strategy? To answer this question we examine the situation where we start from a situation where p=0 and we want to know if a mutant with a higher value of p could invade. Using the same $\varphi_{m}\left(A\right)$ function defined above we show that:

$$S_{\varphi ,p}\propto \left({\hat{v}}_{L}-{\hat{v}}_{V}B\right)\left(F_{m}\left(\hat{A}\right)-{\varphi_{0}}^{\text{∗}}\right)$$

where ${\varphi_{0}}^{*}$ is the evolutionary stable lysogenisation rate when p=0. If the virus adopts this evolutionary stable strategy and $0<{\varphi_{0}}^{*}<1$ the individual reproductive values of the virus will verify ${\hat{v}}_{L}-{\hat{v}}_{V}B=0$ and, consequently, $S_{\varphi ,p}=0$. In other words, in a constant environment, if the virus has evolved toward the fixed evolutionary stable lysogenisation strategy there is no selection for plasticity. However, we will see in the next section that a mutation that affects plasticity can invade the fixed evolutionary stable lysogenisation strategy because a conditional strategy allows the virus to better cope with the periodic fluctuations of the environment.

In a fluctuating environment, we need to compute numerically the selection gradient $S_{\varphi ,\varphi_{0}}\left(t\right)$ given above. In the absence of plasticity (p=0) the evolution of a fixed lysogenisation strategy $\varphi_{0}^{*}$ verifies the following condition:

$$\left\langle S_{\varphi ,\varphi_{0}}\left(t\right)\right\rangle =\langle \left(v_{L}\left(t\right)-Bv_{V}\left(t\right)\right)abS\left(t\right)f_{V}\left(t\right)\rangle =0$$

Can a mutant with a higher value of p invade? The selection gradient on the trait p is equal to:

$$\left\langle S_{\varphi ,p}\left(t\right)\right\rangle =\langle \left(v_{L}\left(t\right)-Bv_{V}\left(t\right)\right)abS\left(t\right)f_{V}\left(t\right)\left(F_{m}\left(A\right)-\varphi_{0}^{*}\right)\rangle$$

Using $\langle S_{\varphi ,\varphi_{0}}\left(t\right)\rangle =0$ this selection gradient reduces to:

$$\left\langle S_{\varphi ,p}\left(t\right)\right\rangle =Cov\left(X_{\varphi }\left(t\right),F_{m}\left(A\right)\right)$$

where:

$$X_{\varphi }\left(t\right)=\left(v_{L}\left(t\right)-Bv_{V}\left(t\right)\right)abS\left(t\right)f_{V}\left(t\right)$$

In other words, selection for plasticity is governed by the sign of the covariance between the quantities $X_{\varphi }\left(t\right)$ and $F_{m}\left(A\right)$. Plasticity will be selected for if the functions $F_{m}\left(A\right)$ can track the fluctuations of $X_{\varphi }\left(t\right)$ and generate a positive covariance between $X_{\varphi }\left(t\right)$ and $F_{m}\left(A\right)$. In particular we show that evolutionary stable plastic strategies typically evolve a positive value of $\lambda_{\varphi }$, the slope of the function $F_{m}\left(A\right)$ when $F_{m}\left(A\right)=\varphi_{max}/2$ (Figure S2). Figure S2 also shows that the evolutionary stable plastic strategy (when we allow $A_{\varphi }$ to evolve freely) can invade the fixed evolutionary stable strategy.

#### Evolution of reactivation

Similarly, the selection on reactivation is driven by the following gradient of selection (where z is the evolving trait that can affect the reactivation rate α):

$$S_{\alpha ,z}\left(t\right)=\left(Bv_{V}\left(t\right)-v_{L}\left(t\right)\right){\frac{\partial \alpha_{m}\left(A\right)}{\partial z_{m}}|}_{z_{m}=z}f_{L}\left(t\right)$$

In other words, selection for reactivation may vary with time and the direction of selection is governed by the difference between the reproductive value $v_{V}\left(t\right)$ of each of the B virions produced upon lysis and $v_{L}\left(t\right)$ the reproductive value of a prophage (note the opposite sign between $S_{\varphi ,z}\left(t\right)$ and $S_{\alpha ,z}\left(t\right)$). The long-term evolution of the shape of the reaction norm α(A) is driven by the integral of this instantaneous selection gradient over a period of the fluctuation of the environment:

$$S_{\alpha ,z}=\langle S_{\alpha ,z}\left(t\right)\rangle$$

Moving forward in the analysis of the evolution of reactivation requires an explicit function $\alpha_{m}\left(A\right)$ and we use:

$$\alpha_{m}\left(A\right)=\alpha_{0m}\left(1-p_{m}\right)+p_{m}G_{m}\left(A\right)$$

with

$$G_{m}\left(A\right)=\alpha_{max}/\left(1+{\text{e}}^{-\lambda_{\alpha }\left(A\left(t\right)-A_{\alpha }\right)}\right)$$

This function allows us to consider lysogenisation as a fixed strategy $\alpha_{0}$ when p=0 (no plasticity) or as a conditional function (plastic trait) where $\alpha_{max}$ is the maximal value of $G_{m}\left(A\right)$, $A_{\alpha }$ is the value of A where $G_{m}\left(A\right)=\alpha_{max}/2$ and $\lambda_{\alpha }$ is the slope of the function $G_{m}\left(A\right)$ when $A=A_{\alpha }$.

Let us first consider the evolution of a fixed lysogenisation strategy (when p=0) in a constant environment. When the influx of susceptible bacteria does not vary with time the selection on the trait $\alpha_{0}$ is given by:

$$S_{\alpha ,\alpha_{0}}\propto \left({\hat{v}}_{V}B-{\hat{v}}_{L}\right){\hat{f}}_{L}$$

The sign of $S_{\alpha ,\alpha_{0}}$ is driven by the sign of $\left({\hat{v}}_{V}B-{\hat{v}}_{L}\right)$, where the hat symbol refers to the value of the dynamical variables at this endemic equilibrium. We thus need to determine the reproductive values at this endemic equilibrium.

Following the same argument as above for the evolution of lysogenisation we know that if $\theta_{0}=0$ then ${\hat{v}}_{V}B-{\hat{v}}_{L}<0$. In other words, selection favors mutations that decrease the rate of reactivation α. In our model, we find that this influx of susceptible cells has to be higher than a threshold for reactivation to evolve: $\theta_{0}>\;ab\hat{V}\hat{S}$.

Could an evolutionary stable fixed strategy be invaded by a plastic strategy? To answer this question we examine the situation where we start from a situation where p=0 and we want to know if a mutant with a higher value of p could invade. Using the same $\alpha_{m}\left(A\right)$ function defined above we show that:

$$S_{\alpha ,\alpha_{0}}\propto \left({\hat{v}}_{V}B-{\hat{v}}_{L}\right)\left(G_{m}\left(\hat{A}\right)-{\alpha_{0}}^{\text{∗}}\right)$$

where ${\alpha_{0}}^{*}$ is the evolutionary stable lysogenisation rate when p=0. If the virus adopts this evolutionary stable strategy and $0<{\alpha_{0}}^{*}<1$ the individual reproductive values of the virus will verify ${\hat{v}}_{V}B-{\hat{v}}_{L}=0$ and consequently $S_{\alpha ,p}=0$. In other words, in a constant environment, if the virus has evolved toward the fixed evolutionary stable reactivation strategy there is no selection for plasticity. However, as pointed out above for the evolution of lysogenisation, we will see in the next section that a mutation that affects plasticity can invade the fixed evolutionary stable reactivation strategy because a conditional strategy allows the virus to better cope with the periodic fluctuations of the environment.

In a fluctuating environment, we need to compute numerically the selection gradient $S_{\alpha ,\alpha_{0}}\left(t\right)$ given above. In the absence of plasticity (p=0) the evolution of a fixed reactivation strategy ${\alpha_{0}}^{*}$ verifies the following condition:

$$\left\langle S_{\alpha ,\alpha_{0}}\left(t\right)\right\rangle =\langle \left(Bv_{V}\left(t\right)-v_{L}\left(t\right)\right)f_{L}\left(t\right)\rangle =0$$

Can a mutant with a higher value of p invade? The selection gradient on the trait p is equal to:

$$\left\langle S_{\alpha ,p}\left(t\right)\right\rangle =\left(Bv_{V}\left(t\right)-v_{L}\left(t\right)\right)f_{L}\left(t\right)\left(G_{m}\left(A\right)-\alpha_{0}^{*}\right)$$

Using $\langle S_{\alpha ,\alpha_{0}}\left(t\right)\rangle =0$ this selection gradient reduces to:

$$\left\langle S_{\alpha ,p}\left(t\right)\right\rangle =Cov\left(X_{\alpha }\left(t\right),G_{m}\left(A\right)\right)$$

where:

$$X_{\alpha }\left(t\right)=\left(Bv_{V}\left(t\right)-v_{L}\left(t\right)\right)f_{L}\left(t\right)$$

In other words, selection for plasticity is governed by the sign of the covariance between the quantities $X_{\alpha }\left(t\right)$ and $G_{m}\left(A\right)$. Plasticity will be selected for if the functions $G_{m}\left(A\right)$ can track the fluctuations of $X_{\alpha }\left(t\right)$ and generate a positive covariance between $X_{\alpha }\left(t\right)$ and $G_{m}\left(A\right)$. In particular we show that evolutionary stable plastic strategies typically evolve a negative value of $\lambda_{\alpha }$, the slope of the function $G_{m}\left(A\right)$ when $G_{m}\left(A\right)=\alpha_{max}/2$ (Figure S2). Figure S2 also shows that the evolutionary stable plastic strategy (when we allow $A_{\alpha }$ to evolve freely) can invade the fixed evolutionary stable strategy ${\alpha_{0}}^{*}$.

#### Coevolution of lysogenisation and reactivation

Lysogenisation and reactivation are expected to evolve jointly to respond to a fluctuation in arbitrium and the selection gradients $S_{\varphi ,z}\left(t\right)$ and $S_{\alpha ,z}\left(t\right)$ can be used to identify the ultimate coevolutionary outcomes between these two plastic traits. Figure S3 shows the direction of selection when both $A_{\varphi }$ and $A_{\alpha }$ are allowed to coevolve. This figure allows us to identify a coevolutionary strategy (the black dot) where ${A_{\varphi }}^{*}>{A_{\alpha }}^{*}$ (see also Figure 1).

#### Experimental Methods

##### Construction of phage deletion mutants

Phi3T deletion mutants were constructed using the pJOE8999 vector, which contains a single guide RNA sequence and *cas9* under the control of a mannose-inducible promoter^31^. sgRNA targeting the region to be deleted were ligated into the BsaI digested vector (Table S2). 750bp flanking regions of each deletion were introduced into the sfiI-digested vector using NEBuilder HiFi DNA Assembly Master Mix (Table S2). The resulting constructs were transformed into *E.coli* DH5α cells for amplification before transformation into *Bacillus subtilis 168* harboring a phi3T lysogen to generate the desired deletion mutants^32^. Cells were screened using PCR to identify cells containing the deletion. For construction of phi3T^*aimR*-N202A^, a gBlock of the phi3T *aimR* gene with residue 202 mutated from asparagine to alanine, and a 750bp flanking region were introduced to the vector as described above. The mutation from asparagine to alanine abolishes the signal peptide-binding capacity of the receptor but does not interfere with its ability to activate *aimX* expression.

##### Prophage reactivation

To quantify prophage reactivation, we picked individual colonies of lysogens into 6ml of LB media and incubated shaking overnight at 37°C and 200rpm. Overnight cultures of lysogens were washed 4x in 1xM9 salts to remove phage and resuspended in fresh LB media. They were diluted to ∼4x10^5^ cells/mL in LB media or LB media containing various signaling peptide concentrations and incubated for 8 or 18 hours at 37°C and 200rpm. 100μl of culture was sampled into chloroform and centrifuged for 10min at 3500 g. Small-drop plaque assays were used to calculate sample PFU/mL. Log-phase cultures of BEST7003:aimX were mixed with LB media supplemented with 0.2% xylose, 0.1mM MnCl_2_, 5mM MgCl_2_ and 0.75% agar, and added to LB agar plates containing 0.1mM MnCl_2_ and 5mM MgCl_2_. Phage-containing supernatant was serially diluted and 10μl spotted onto bacterial lawns. Plates were incubated overnight at 37°C and the number of PFU/mL calculated.

For testing the effects of *aimX* expression on prophage reactivation, *Bacillus subtilis* BEST7003:*aimX* was lysogenised with phi3TΔ*aimP*. Conditions were as described above but lysogens cultures were diluted to ∼4x10^5^ cells/mL in LB media (uninduced) or LB media supplemented with 0.2% xylose (induced).

##### Lysogen formation

To quantify lysogen formation at different signal concentrations, we diluted overnight cultures of *Bacillus subtilis* BEST7003 1:100 into 6ml LB media and incubated until they reached and OD_600_ of ∼0.2. Cultures were diluted to ∼4x10^5^ cells/mL in LB media containing 0.1mM MnCl_2_, 5mM MgCl_2_ and concentrations of signaling peptide ranging from 0nM to 500nM. In order to ensure native signal production did not interfere with signal concentrations in the media, samples were infected with Phi3T*ΔaimP(spc),* a mutant carrying a spectinomycin resistance cassette in place of the *aimP* gene. Hosts were infected to an MOI of 0.1 and incubated for 40 minutes at 37°C to allow for phage absorption and expression of the spectinomycin resistance cassette. Samples were serially diluted and plated onto LB agar containing 100μg/mL spectinomycin to quantify lysogens/mL.

##### Quantification of Signal Decay

We used the signal reporter strain BEST7003:RPXgfp, a gift from Rotem Sorek, to quantify the concentration of signaling peptide in a given media. The signal reporter contains the phi3T *AimR-AimP-AimX* locus genetically fused to a fluorescent reporter gene (gfp) which has been inserted into the host *amyE* locus, and when grown in media containing signaling peptide, expresses GFP in a concentration-dependent manner. Maximum fluorescence is observed in the absence of signal and is gradually repressed as the concentration of signaling peptide increases. The signal concentration in a given media was quantified by comparison with a calibration curve of spent media supplemented with known signal peptide concentrations. To ensure the calibration and unknown samples differ only in the signal peptide concentration, and to account for the effect of spent media on the reporter, calibration curve spent medium was always obtained in parallel experiments using the same growth conditions but lacking signal peptide. Percentage signal decay was calculated as ((T_0_ Signal Concentration – T_x_ Signal Concentration)/ T_0_ Signal Concentration))^∗^100.

To quantify signal decay in LB media, we incubated 10ml of LB media at a signal concentration of 1000nM in 15ml falcon tubes at 37°C. For calibration curve spent media, we incubated LB media without signal under the same conditions. After 24hrs, 48hrs or 72hrs, we mixed sample media 1:1 with fresh LB. To construct a calibration curve, known concentrations of signaling peptide were added to the calibration curve spent media and mixed 1:1 with fresh LB. Washed overnight cultures of BEST7003:RPXgfp were diluted 1:100 into the sample and calibration curve spent medium, and OD_600_ and GFP fluorescence (488 nm excitation/518 nm emission) were quantified over 7 hours at 37°C in a BioTek plate reader. A quadratic model was fitted to stationary-phase (6hr) fluorescence values of the calibration curve and the signal concentration of unknown samples calculated.

To quantify signal decay in the presence of host cells, we picked individual colonies of *Bacillus subtilis* 3610 and 3610Δ*oppD* into 6ml LB media and incubated them overnight at 37°C and 200rpm. Overnight cultures of each strain were washed 3x in 1xM9 salts and resuspended in fresh LB. They were diluted to ∼4x10^5^ cells/mL in LB containing 1000nM of signaling peptide and incubated for 18 hours at 37°C and 200rpm. For calibration curve spent media, culturing conditions were identical except cells were cultured in LB lacking signaling peptide. After 18 hours, we removed cells by passing cultures through a 0.22 μm filter and removed large molecules and cellular debris by passing spent media through a 3kDa filter. Known concentrations of signaling peptide were then added to the calibration curve spent media, the media were mixed with the signal reporter as outlined above, and signal concentrations calculated from calibration curves.

To quantify signal decay by secreted extracellular products, we picked individual colonies of *Bacillus subtilis* BEST7003 into 6ml LB media and incubated them overnight at 37°C and 200rpm. Overnight cultures were washed 3x in 1xM9 salts and resuspended in fresh LB. They were diluted to ∼4x10^5^ cells/mL and incubated at 37°C and 200rpm. After 3 hours (relatively low-density) and 8 hours (relatively high density) we removed cells by centrifuging (3500 g, 10 m) and passing cultures through a 0.22μm filter. We supplemented low density and high density spent media to 1000nM with signaling peptide and incubated the media at 37°C for 12 hours. For calibration curve spent media, we incubated the media for 12 hours without signaling peptide. We passed both the sample and calibration curve media through a 3kDa filter to remove any proteases and prevent further signal degradation and known concentrations of signaling peptide were added to the calibration curve spent media. These media were mixed with the signal reporter as outlined above, and signal concentrations calculated from calibration curves.

##### Prophage signal production

To test for signal production by prophage, we picked individual colonies of phi3T and phi3TΔ*aimP* lysogens of BEST7003, and uninfected BEST7003, into 6ml of LB media and incubated shaking overnight at 37°C and 200rpm. Overnight cultures were washed 4x in 1xM9 salts and resuspended in fresh LB media. They were diluted to ∼1x10^6^ cells/mL in LB media and incubated for 2 hours at 37°C and 200rpm. After 2 hours we removed cells by centrifuging (3500 g, 10min) and passing cultures through a 0.22μm filter before passing spent media through a 3kDa filter to remove cellular debris and large metabolites. Spent media was mixed 1:1 with fresh LB, and overnight cultures of phi3TΔ*aimP* lysogens washed 4x in 1xM9 salts were added to ∼4x10^5^ cells/mL. Lysogen cultures were incubated for 8 hours at 37°C and 200rpm before 100μl of culture was sampled into chloroform and centrifuged for 10 m at 3500 g. Small-drop plaque assays were performed as described above and prophage induction calculated as PFU/mL.

### Quantification and statistical analysis

We carried out all statistical analyses in the R statistical environment^26^ (v3.3.3, http://www.R-project.org). Except where stated, we carried out standard analyses (T-Test, Linear models, etc.) assuming normal errors.

## Data Availability

Data have been deposited at the Dryad data repository and are publicly available as of the date of publication. DOIs are listed in the Key resources table.

## Supplemental information

Document S1. Figures S1–S4 and Tables S1 and S2

Document S2. Article plus supplemental information
