# Supplementary material for: Regulation of prophage induction and lysogenization by phage communication systems
Source: Curr Biol. 2021 Nov 22;31(22):5046–5051.e7. doi: 10.1016/j.cub.2021.08.073 (PMC8612742; doi:10.1016/j.cub.2021.08.073)
Supplement: Document S1. Figures S1–S4 and Tables S1 and S2 [file mmc1.pdf]

Current Biology, Volume *31*

Supplemental Information

Regulation of prophage induction and lysogenization by phage communication systems

John B. Bruce, Sébastien Lion, Angus Buckling, Edze R. Westra, and Sylvain Gandon

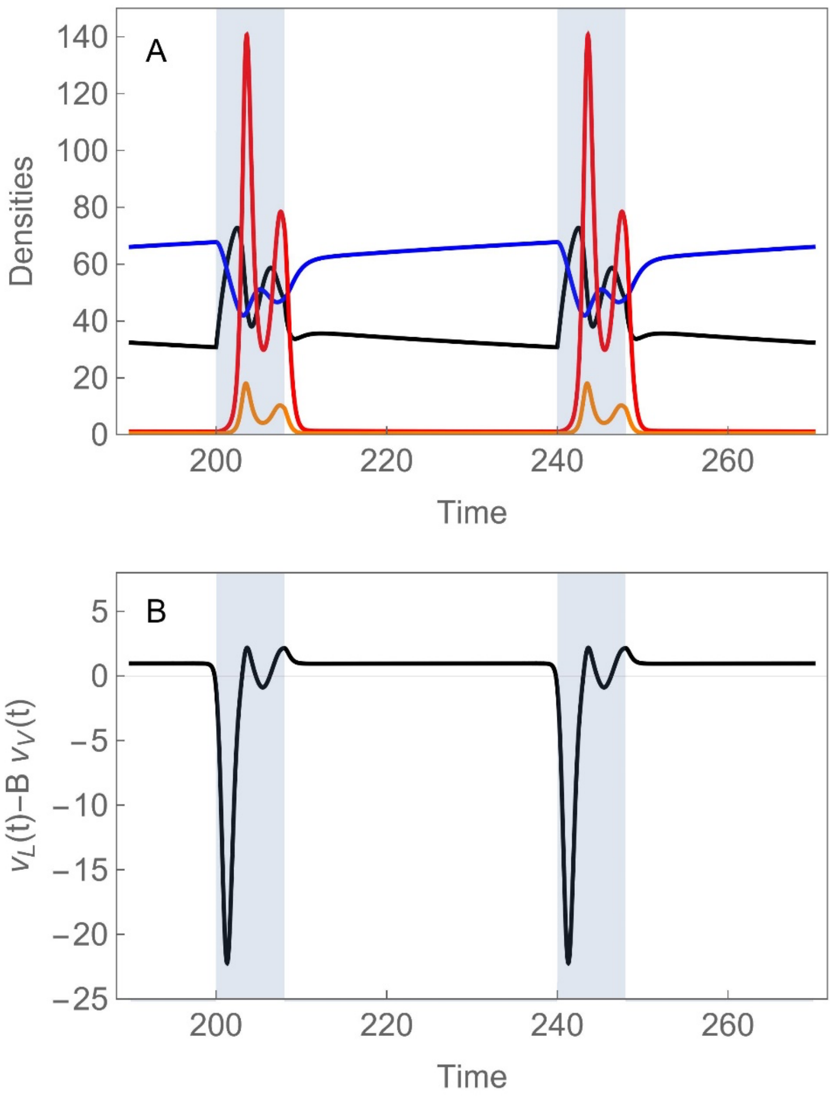

Figure S1: Epidemiological dynamics and dynamics of reproductive values – related to Figure 1.

In (A) we plot the temporal dynamics of the densities of susceptible cells (in black), lysogenized cells (blue), virus particles (red) and arbitrium concentration (orange). In this scenario we allow θ to fluctuate periodically as a square wave between $\theta_{min} = 0$ (for 80% of the period) and $\theta_{max} = 30$ (for 20% of the period) with a period $T = 40$. The time points where $\theta(t) = \theta_{max}$ are indicated with the gray shading. In (B) we plot the temporal dynamics of $v_L(t) - Bv_V(t)$ which drives the evolution of plasticity. Note that the concentration of arbitrium increases when the density of susceptible cells starts to drop. Other parameter values: $r = 1$, $a = 0.1$, $b = 0.1$, $\kappa = 0.01$, $d = 0.01$, $B = 20$, $d_V = 0.1$, $d_A = 10$, $\delta = 0.1$, $\pi_V = 5$, $\pi_L = 0.1$, $p = 0$, ${\phi_0}^* = 0.026$, ${\alpha_0}^* = 0.003$.

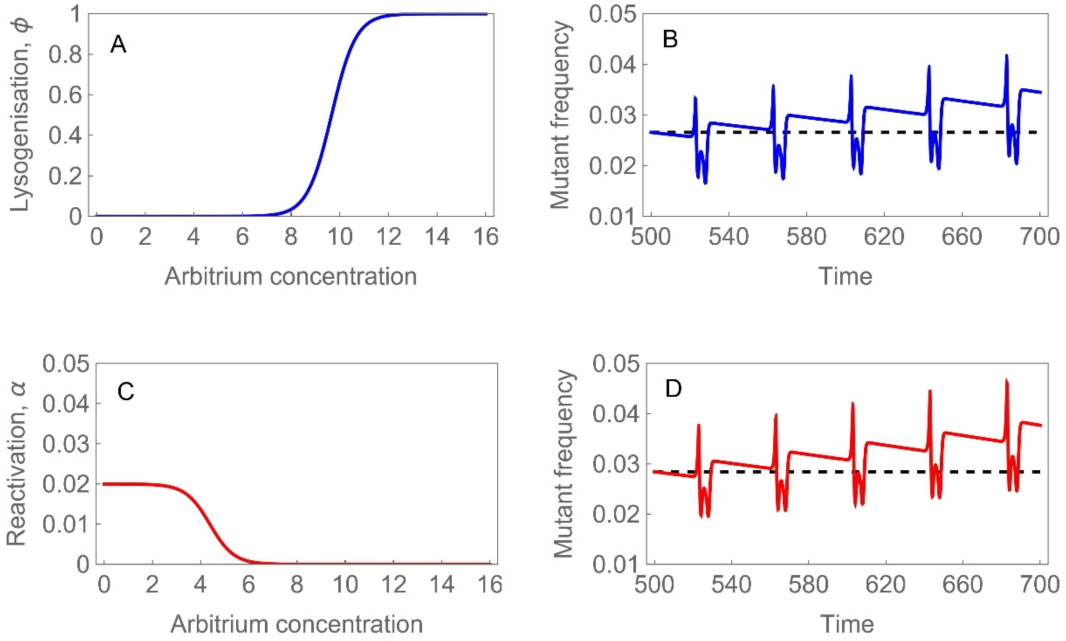

Figure S2: Evolution of plastic lysogenisation (panels A and B) and plastic reactivation (panels C and D) – related to Figure 1.
We used the gradient of selection (10) to identify the evolutionary stable plastic strategy (when $p = 1$) for the trait A_ϕ which refers to the threshold value of the arbitrium concentration above which lysogenisation is triggered (${A_\phi}^* = 9.66$). In (A) we plot the evolutionary stable plastic lysogenisation strategy as a function of arbitrium concentration. In (B) we show that a mutant which adopts some plasticity ($p_m = 0.1$) can invade a resident strategy that adopts an evolutionary stable strategy with no plasticity ($p = 0$, ${\phi_0}^* = 0.026$, ${\alpha_0}^* = 0.003$). We used the gradient of selection (18) to identify the evolutionary stable plastic strategy (when $p = 1$) for the trait A_α which refers to the threshold value of the arbitrium concentration below which reactivation of the prophage is triggered (${A_\alpha}^* = 4.38$). In (C) we plot the evolutionary stable plastic reactivation strategy as a function of arbitrium concentration. In (D) we show that a mutant which adopts some plasticity ($p_m = 0.1$) can invade a resident strategy that adopts an evolutionary stable strategy with no plasticity ($p = 0$, ${\phi_0}^* = 0.026$, ${\alpha_0}^* = 0.003$). Note that even if the mutant is selected over one period of the fluctuation, its frequency may transiently drop when the instantaneous gradient of selection is negative. In this scenario we allow θ to fluctuate periodically as a square wave between $\theta_{min} = 0$ (for 80% of the period) and $\theta_{max} = 30$ (for 20% of the period) with a period $T = 40$. The dashed line is the frequency of the mutant at time $t = 500$. Other parameter values: $r = 1$, $a = 0.1$, $b = 0.1$, $\kappa = 0.01$, $d = 0.01$, $B = 20$, $d_V = 0.1$, $d_A = 10$, $\delta = 0.1$, $\pi_V = 5$, $\pi_L = 0.1$, $\phi_{max} = 1$, $\lambda_\phi = 2$, $\alpha_{max} = 0.02$, $\lambda_\alpha = -2$, $A_\alpha = 2$.

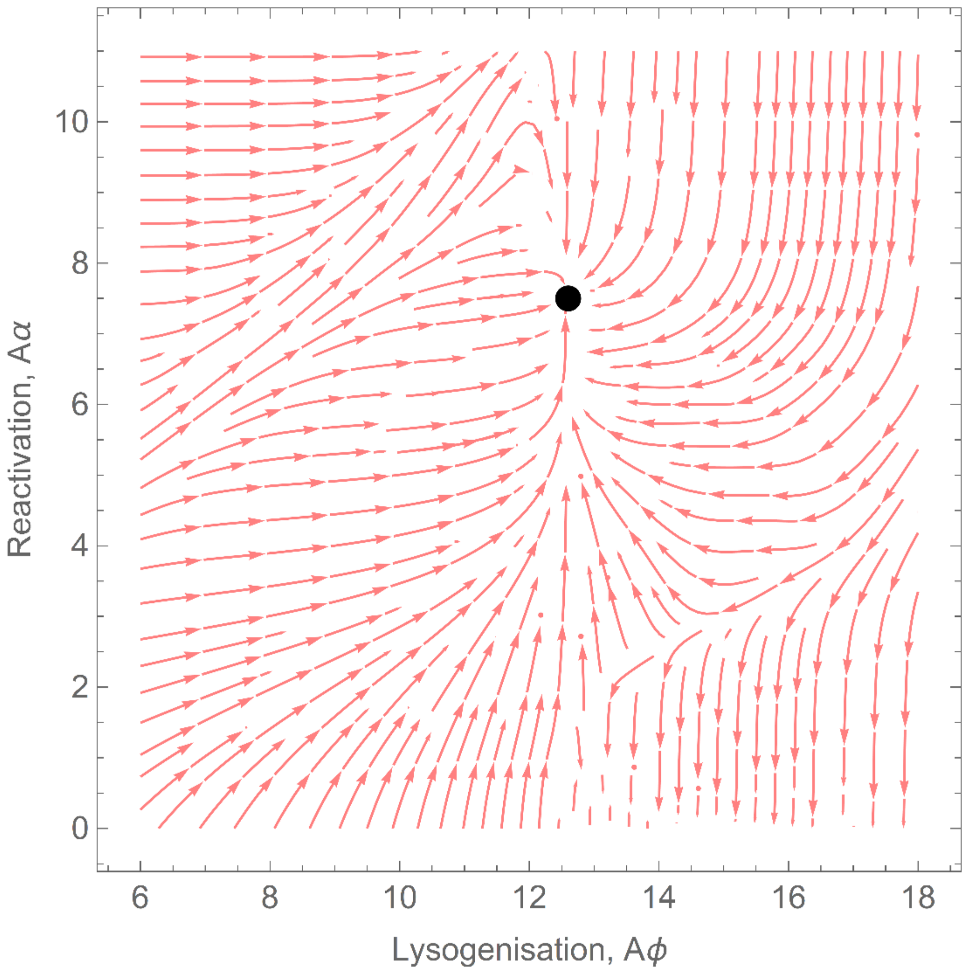

Figure S3: Coevolution of plastic lysogenisation and reactivation strategies – related to Figure 1.

We used the gradients of selection (10) and (18) to identify the coevolutionary stable plastic strategy (black dot) when $p = 1$ for the traits A_ϕ and A_α (${A_\phi}^* = 12.6$ and ${A_\alpha}^* = 7.5$, see also **Figure 1** in the main text). In this scenario we allow θ to fluctuate periodically as a square wave between $\theta_{min} = 0$ (for 80% of the period) and $\theta_{max} = 40$ (for 20% of the period) with a period $T = 60$. Other parameter values: $r = 0$, $a = 0.1$, $b = 0.1$, $\kappa = 0.01$, $d = 0.01$, $B = 20$, $d_V = 0.1$, $d_A = 10$, $\delta = 0.1$, $\pi_V = 5$, $\pi_L = 0.1$, $\phi_{max} = 1$, $\lambda_\phi = 2$, $\alpha_{max} = 0.02$, $\lambda_\alpha = -2$.

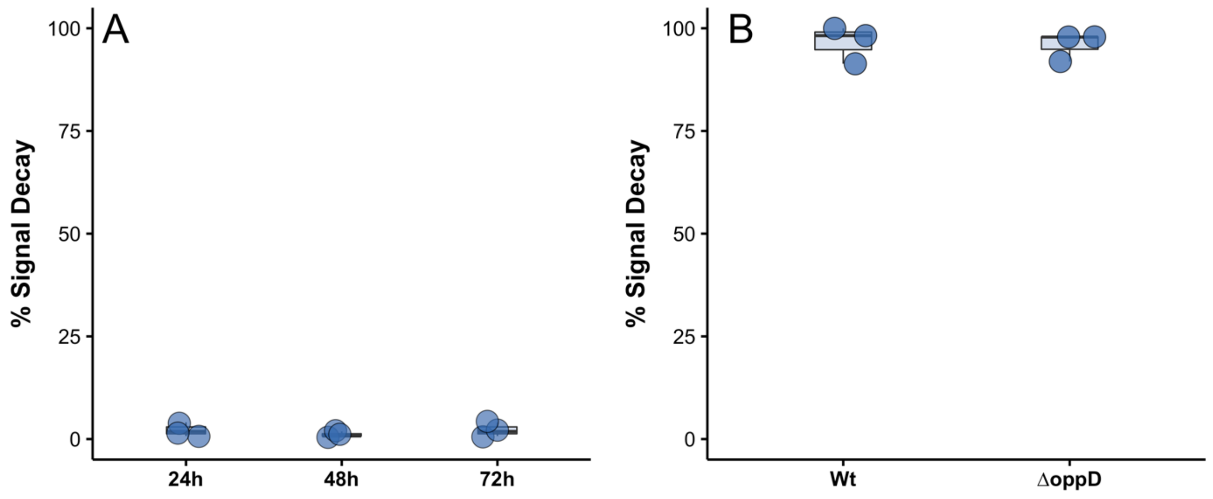

Figure S4. **Signal Decay – related to figure 4.**
(A) LB media supplemented with signalling peptide (1000nM) was incubated in the absence of bacterial cells at 37C and signal decay quantified after 24, 48 and 72 hours (n=3). **(B)** LB media supplemented with signalling peptide (1000nM) was incubated with *Bacillus subtilis* 3610 or 3610ΔoppD (a mutant unable to internalise signalling peptide) and decay quantified from spent media after 18 hours (n=3).

Dynamical variables and parameters	Definitions
$S(t)$ and $L(t)$	density of susceptible and lysogenic bacteria<br>$N(t) = S(t) + L(t)$
$V(t)$	density of virus particles
$A(t)$	concentration of arbirtrium
$\theta(t)$	influx of susceptible bacteria
r	growth rate of bacterial cells
$v_V(t)$ and $v_L(t)$	individual reproductive values of the virus in the free virus particle stage (V) or in the lysogenic stage (L)
κ	density-dependent coefficient
d	mortality rate of bacterial cells
B	viral burst size
a	adsorption constant
b	probability of infection after adsorption
ϕ	probability of lysogenisation<br>(may be a function of arbitrium concentration)
α	rate of reactivation<br>(may be a function of arbitrium concentration)
d_V	mortality rate of virus particles
π_V and π_L	production rate of arbitrium trough lysis and lysogeny
d_A	degradation rate of arbitrium
δ	degradation rate of arbitrium by bacterial cells
$\mathcal{S}_{X,Y}(t)$	instantaneous gradient of selection on a mutation that affects the parameter Y which governs the life-history trait X

Table S1: Main parameters and dynamical variables of the model – related to STAR methods

**Identifier**	**Oligo**	**Source**	**Function**
ΔP_up_F	5'-ctcactatagggtcgacggccaacgtttcaaatgcaatgctgatg-3'	IDT	phi3TΔaimP Upstream Flanking Region
ΔP_up_R	5'-ttcaattatttaactaaatagagataaggtttaataattcaag-3'	IDT	phi3TΔaimP Upstream Flanking Region
ΔP_dwn_F	5'-atctctatttagttaaataattgaataggtaatacataatactatc-3'	IDT	phi3TΔaimP Downstream Flanking Region
ΔP_dwn_R	5'-atttcttaatctagaaaggccttattctttttattttcctcagtataatatttattg-3'	IDT	phi3TΔaimP Downstream Flanking Region
ΔR_up_F	5'-ctcactatagggtcgacggccaacgtgaatcgtcaaaaattgtatag-3'	IDT	phi3TΔaimR Upstream Flanking Region
ΔR_up_R	5'-aatttgtcaaacttaatcctcattgtgcataattg-3'	IDT	phi3TΔaimR Upstream Flanking Region
ΔR_dwn_F	5'-caatgaggattaagtttgacaaatttgaaaggag-3'	IDT	phi3TΔaimR Downstream Flanking Region
ΔR_dwn_R	5'-atttcttaatctagaaaggccttatatatgaagattaacacgagtaaag-3'	IDT	phi3TΔaimR Downstream Flanking Region
ΔRPX_up_F	5'-ctcactatagggtcgacggccaacgtgaatcgtcaaaaattgtatag-3'	IDT	phi3TΔaimRPX Upstream Flanking Region
ΔRPX_up_R	5'-ttatttttattctttaatcctcattgtgcataattg-3'	IDT	phi3TΔaimRPX Upstream Flanking Region
ΔRPX_dwn_F	5'-aatgaggattaaagaataaaaataaaattaattattgcataatc-3'	IDT	phi3TΔaimRPX Downstream Flanking Region
ΔRPX_dwn_R	5'-atttcttaatctagaaaggccttataccaatcagtatcttgcttaaag-3'	IDT	phi3TΔaimRPX Downstream Flanking Region
phi3TAimR-N202A _up_F	5'-ctcactatagggtcgacggccaacgtaaagaaaacaaatagagaagtg-3'	IDT	phi3TAimR-N202A Upstream Flanking Region
phi3TAimR-N202A _up_R	5'-tgattccctaatagcatcatttaaactgatttc-3'	IDT	phi3TAimR-N202A Upstream Flanking Region
phi3TAimR-N202A _gBlock_F	5'-tttaaatgatgctattagggaatcagggaaatg-3'	IDT	phi3TAimR-N202A gBlock
phi3TAimR-N202A _gBlock_R	5'-atttcttaatctagaaaggccttatctaaatagagataaggtttaataattcaag-3'	IDT	phi3TAimR-N202A gBlock
ΔX_up_F	5'-ctcactatagggtcgacggccaacgaaaatagcctacttgaagc-3'	IDT	phi3TΔaimX Upstream Flanking Region
ΔX_up_R	5'-ttatttttattctttaagcaccacgaattgc-3'	IDT	phi3TΔaimX Upstream Flanking Region
ΔX_dwn_F	5'-cgtggtgcttaaagaataaaaataaaattaattattgcataatc-3'	IDT	phi3TΔaimX Downstream Flanking Region
ΔX_dwn_R	5'-atttcttaatctagaaaggccttataccaatcagtatcttgcttaaag-3'	IDT	phi3TΔaimX Downstream Flanking Region
sg_Phi3T_aimP_F	5'-tacgTTTTGGTTTAGTAATTCTTA-3'	IDT	aimP sgRNA
sg_Phi3T_aimP_R	5'-aaacTAAGAATTACTAAACCAAAA-3'	IDT	aimP sgRNA
sg_Phi3T_aimR_F	5'-tacgAATTTGATGATTTACCCGAA-3'	IDT	aimR sgRNA
sg_Phi3T_aimR_R	5'-aaacTTCGGGTAAATCATCAAATT-3'	IDT	aimR sgRNA
sg_Phi3T_aimX_F	5'-tacgCAATTCAATAATTGCTCAAG-3'	IDT	aimX sgRNA
sg_Phi3T_aimX_R	5'-aaacCTTGAGCAATTATTGAATTG-3'	IDT	aimX sgRNA

Table S2: Oligos – related to STAR methods
